# Plasma BCAA concentrations during exercise of varied intensities in young healthy men—the impact of endurance training

**DOI:** 10.7717/peerj.10491

**Published:** 2020-12-21

**Authors:** Anna Gawedzka, Marcin Grandys, Krzysztof Duda, Justyna Zapart-Bukowska, Jerzy A. Zoladz, Joanna Majerczak

**Affiliations:** 1 Department of Muscle Physiology, Institute of Basic Sciences, Faculty of Rehabilitation, University School of Physical Education in Krakow, Krakow, Poland; 2 Department of Biochemical Analytics, Faculty of Pharmacy, Jagiellonian University, Medical College, Krakow, Poland; 3 Institute of Health Care, State Higher Vocational School in Tarnow, Tarnow, Poland; 4 Department of Neurobiology, Faculty of Health Sciences, Poznan University School of Physical Education in Poznan, Poznan, Poland

**Keywords:** BCAA, Incremental exercise, Endurance training, Maximal oxygen uptake

## Abstract

**Background:**

Branched-chain amino acids (BCAA) i.e., leucine (Leu), isoleucine (Ile) and valine (Val) are important amino acids, which metabolism play a role in maintaining system energy homeostasis at rest and during exercise. As recently shown lowering of circulating BCAA level improves insulin sensitivity and cardiac metabolic health. However, little is known concerning the impact of a single bout of incremental exercise and physical training on the changes in blood BCAA. The present study aimed to determine the impact of a gradually increasing exercise intensity—up to maximal oxygen uptake (VO_2max_) on the changes of the plasma BCAA [∑BCAA]_pl_, before and after 5-weeks of moderate-intensity endurance training (ET).

**Methods:**

Ten healthy young, untrained men performed an incremental cycling exercise test up to exhaustion to reach VO_2max_, before and after ET.

**Results:**

We have found that exercise of low-to-moderate intensity (up to ∼50% of VO_2max_ lasting about 12 min) had no significant effect on the [∑BCAA]_pl_, however the exercise of higher intensity (above 70% of VO_2max_ lasting about 10 min) resulted in a pronounced decrease (*p* < 0.05) in [∑BCAA]_pl_. The lowering of plasma BCAA when performing exercise of higher intensity was preceded by a significant increase in plasma lactate concentration, showing that a significant attenuation of BCAA during incremental exercise coincides with exercise-induced acceleration of glycogen utilization. In addition, endurance training, which significantly increased power generating capabilities at VO_2max_ (*p* = 0.004) had no significant impact on the changes of [∑BCAA]_pl_ during this incremental exercise.

**Conclusion:**

We have concluded that an exercise of moderate intensity of relatively short duration generally has no effect on the [∑BCAA]_pl_ in young, healthy men, whereas significant decrease in [∑BCAA]_pl_ occurs when performing exercise in heavy-intensity domain. The impact of exercise intensity on the plasma BCAA concentration seems to be especially important for patients with cardiometabolic risk undertaken cardiac rehabilitation or recreational activity.

## Introduction

Amino acids in human body are present mainly in the form of proteins, most of which are contained in skeletal muscles (40–45% of total body protein pool), whereas the pool of free amino acids constitutes less than 2% of total body amino acids. Among the essential amino acids (which cannot be synthesized from other metabolites in human body), the branched-chain amino acids (BCAA) i.e., leucine (Leu), isoleucine (Ile) and valine (Val), are the most relevant amino acids metabolized during exercise (Wagenmakers et al., 1989). Interestingly, circulating BCAA constitute also a biomarker of cardiometabolic diseases (Batch et al., 2013; White & Newgard, 2019). As suggested recently, BCAA by promoting lipid storage in liver and in skeletal muscles contribute to insulin resistance (White & Newgard, 2019). The genetic association studies by *Lotta et al. (2016)* shown strong relationship between higher circulating level of BCAA and type 2 diabetes in humans. In addition, *Wang et al. (2011)* found that five circulating branched-chain and aromatic amino acids (including leucine, isoleucine, valine, tyrosine and phenylalanine) might be used as early markers of diabetes risk. On the other hand, restriction of dietary BCAA by lowering of circulating BCAA levels improves whole-body and skeletal muscle insulin sensitivity in obese rats (White et al., 2016) and lowers lipid accumulation in their hearts, which has positive impact on cardiometabolic health *(McGarrah et al., 2020)*.

It is well known that regular exercise exerts beneficial effects on the human general health including an improvement of insulin sensitivity *(Holloszy, 2005)*. Since skeletal muscle accounts for about 60–80% of postprandial insulin-mediated glucose uptake (Meyer et al., 2002; Soulage et al., 2018), muscle insulin resistance play a key role in the disturbances in glucose homeostasis. Considering the role of BCAA in the insulin resistance and taking into account that skeletal muscles is a major tissue that utilizes BCAA, BCAA oxidation during exercise might have an important impact on the maintaining glucose homeostasis. Specifically, a lowering of circulating BCAA concentration during exercise, which reflects an increase of the BCAA uptake and an enhancement of BCAA catabolism by skeletal muscles during exercise, decreases the risk of an accumulation of BCAA catabolic intermediates, which are known to be involved in the lipid accumulation contributing to muscle insulin resistance (White & Newgard, 2019). As was recently found, 3-Hydroxyisobutyrate (3-HIB), a catabolic intermediate of valine, is indeed positively correlated with insulin resistance in humans (Nilsen et al., 2020).

During exercise BCAA release from splanchnic bed increases and is accompanied by an elevated BCAA uptake by contracting muscles and by an enhancement of BCAA oxidation therein (Wagenmakers et al., 1989). The mechanism responsible for BCAA oxidation in skeletal muscles is attributed to activation of the branched-chain α-keto acid dehydrogenase (BCKDH) complex –  rate limiting enzyme of branched-chain oxoacid oxidation (Wagenmakers et al., 1989; Van Hall et al., 1996). It is generally believed that BCAA are used as an energy substrate mainly during prolonged exercise *(Blomstrand, Celsing & Newsholme, 1988)* and might provide about 3–6% of the total energy demand *(Gibala, 2007).* A significant decrease in plasma BCAA concentration in bloodstream has been observed after sustained exercise (lasting about 1.5 h) as well as after marathon run *(Blomstrand, Celsing & Newsholme, 1988)*. It has been found that prolonged exercise (90 and 120 min duration) at 50–70% maximal power output increases the percentage of active BCKDH in human skeletal muscle by 2- to 5-fold (Wagenmakers et al., 1989), which suggests greater BCAA usage during prolonged exercise.

However, to our best knowledge no data has been presented so far showing the changes of plasma BCAA concentration in varied exercise intensity domains (moderate vs heavy exercise intensity) during incremental exercise. Incremental exercise tests are commonly applied exercise protocols allowing to study physiological responses to varied exercise intensities and to determine key features of exercise tolerance such as lactate threshold (LT), maximal oxygen uptake (VO_2max_), power output at maximal oxygen uptake (PO_max_) *(Majerczak et al., 2014; Zoladz, Rademaker & Sargeant, 1995)*.

Therefore, in the present study we determined the changes in plasma BCAA concentration during the moderate (up to 50% of VO_2max_) and heavy-intensity (above 70% of VO_2max_) exercise domains. We hypothesized that a strenuous exercise even of short duration is accompanied by a significantly greater decrease in plasma BCAA concentration than low-to-moderate intensity exercise. Moreover, we aim to establish the effect of 5-week moderate-intensity endurance training (often used in rehabilitation or as a part of therapy in patients) on the plasma BCAA concentration in relation to exercise tolerance, since the impact of endurance training on the BCAA concentration reported in previous studies is not clear *(Lamont, McCullough & Kalhan, 1999; McKenzie et al., 2000)*. Gaining the missing knowledge on the impact of physical exercise of varied intensities and training on the circulating BCAA in humans seems to be especially important in view of the studies showing a link between the blood BCAA level and cardiometabolic risk (White & Newgard, 2019).

## Materials & Methods

### Ethical approval

The study was approved by Local Ethical Committee in Krakow (No. 9 KBL/OIL/2009) and performed according to guidelines of the Declaration of Helsinki. All volunteers provided written consent for participation in the study after becoming acquainted with the procedures and purpose of the study.

### Subjects and study design

The studied group consisted of 10 healthy, non-smoking, untrained but recreationally active, young men (mean ± SEM: age 22.3 ± 0.4 years; body mass (BM) 79.9 ± 2.7 kg; height 181.0 ± 2.0 cm; body mass index (BMI) 24.3 ± 0.6 kg m^−2^; VO_2max_ 45.6 ± 1.2 mL kg^−1^ min^−1^), who submitted written consent to participate in the study. Before the experiment, participants were asked to follow a normal mixed diet during three days prior to the exercise test that was composed of ∼55% of carbohydrates, ∼25% of fat and ∼20% of protein, and to avoid caffeinated and alcoholic beverages 48 h before exercise. All participants were informed that lifestyle changes may potentially affect the outcome of this study, therefore, they were instructed to maintain their routine diet and asked not to take any dietary supplements throughout the experiment.

The study started with a medical health examination and basic anthropometric measurements. Blood analysis was performed in each subject including morphological and biochemical parameters (see below). Then, subjects performed incremental exercise up to exhaustion to determine exercise tolerance parameters including lactate threshold, VO_2max_ and PO_max_. During the incremental exercise blood samples were taken to analyze plasma lactate and BCAA concentration. Based on this exercise test, the training intensities were established individually for each subject and the 5-week endurance training of moderate-intensity has started. After the training program, anthropometric measurements, incremental exercise test and blood analyses were repeated (see below).

### Exercise protocol

Subjects performed an incremental exercise on the cycloergometer Jaeger Ergo-Line GmbH and Co KG800s (Bitz, Germany) at pedaling rates of 60 rev min^−1^. The subjects were familiarized with the laboratory conditions and the exercise test about 3 days before the onset of this study. The exercise test was preceded by a 6-minute resting period, after which the power output (PO) was increased by 30 W every 3 min until exhaustion. During exercise test gas exchange parameters were recorded continuously breath-by-breath using the Oxycon Champion, Mijnhardt BV (Bunnik, The Netherlands) starting from the 6 min before the test, up to 6 min after its ending (Majerczak et al., 2010; Zoladz, Rademaker & Sargeant, 1995; Zoladz et al., 1998). The incremental exercise test was performed to determine e.g., VO_2max_, PO_max_ and LT. The following criteria of reaching VO_2max_ during incremental test have been applied: (1) at the end of the test a plateauing in VO_2_ in relation to the increase in the power output was evident, (2) at the end of exercise the heart rate (HR) was close to the age-predicted maximum (i.e., above 95% of predicted HR_max_), (3) the end-exercise plasma lactate concentration ([La^−^]_pl_) reached value above eight mmol L^−1^ and the end-exercise respiratory quotient was above 1.10. The incremental test was performed two days before and two days after five weeks of endurance training.

### Endurance training protocol

Endurance training program was performed on cycloergometers Monark 874 E (Monark Exercise AB, Vansbro, Sweden), four times per week throughout 5 weeks, at the pedaling rates 60 rev min ^−1^. All participants were subjected to two different kinds of training exercise protocols. The first training exercise protocol consisted of continuous endurance cycling at the power output corresponding to 90% of the VO_2_ found at the previously determined lactate threshold. The second (intermittent endurance cycling) consisted of 6 min of unloaded cycling followed by a 3 min exercise bout performed at the power output corresponding to 50% Δ (50% difference between the power output reached at VO_2max_ (PO_max_) and the power output obtained at the LT (PO at LT)), repeated 4 times and finished with 4 min of unloaded cycling. Duration of each training bout equalled 40 min and the scaling of the training intensity was based on the principle that the main training workload should be performed in the moderate-intensity domain i.e., below the LT *(Whipp, 1996)*. In this study over 80% of the training workloads were of moderate intensity i.e., they were performed with an intensity corresponding to about 68% of maximum heart rate (HR_max_) (about 50% of VO_2max_), whereas some of the training workloads (∼15%) were planned to be performed in the heavy-intensity domain i.e., above the LT *(Whipp, 1996)*. The continuous endurance cycling was performed on Tuesdays and Thursdays, and the intermittent endurance cycling on Mondays and Fridays. Each training session was monitored using a heart rate monitor (Polar S810, Finland) and supervised by at least one member of the research team. During the 5 weeks training period, volunteers performed on average 19.8 ± 0.2 training sessions (9.9 ± 0.1 sessions of continuous endurance cycling in the moderate-intensity domain and 9.9 ± 0.1 sessions of intermittent endurance cycling in the heavy-intensity domain), and the total training time amounted to 13.2 ± 0.1 h. Compliance with the training program was excellent and amounted to 99 ± 1% (only one subject missed two training sessions, because of short-term stomach problems). The details of the training program were described previously (Grandys et al., 2009; Majerczak et al., 2010).

### Measurements

#### Anthropometric measurements

Body mass was measured using standard anthropometric equipment (Radwag WPT 150, Radom, Poland), with the participants lightly clothed and shoeless. Body fat percentage was estimated using skinfold thickness measurements at the non-dominant upper arm triceps and subscapular sites. The measurements were performed by the same investigator with a calibrated skinfold caliper to the nearest 0.1 mm. The skinfold value was determined as the logarithm value of the sum of the thickness in mm at the two sites and body fat content was calculated using equation given by *Hassager et al. (1986)*. Body fat percentage can be calculated using this equation with a standard error of estimate (SEE) of 2.2 kg (2.8%).

#### Blood morphology and clinical chemistry parameters analysis

Blood samples for the assessment of basal hematological and biochemical variables were taken in the fasting state from the antecubital vein. In addition, before, during and at the end of incremental exercise blood samples were taken from the antecubital vein (in a fed state, see below) via Abbot Int-Catheter, Ireland (18G/1.2 × 45 mm) for plasma lactate and plasma amino acids determination. Blood for complete blood count (CBC) was collected in tubes containing EDTA, placed in a rocker for 30 min and stored in 4 °C, up to 24 h, until analysis. Blood for parameters analyzed in plasma (pl), that is, glucose, insulin and BCAA, was collected in plain tubes containing EDTA. Blood for plasma lactate [La^−^]_pl_ analysis was collected in tubes containing 1 mg ammonium oxalate and 5 mg sodium fluoride. Blood samples after mixing were centrifuged at 653 *g* for 15 min at 4 °C. Blood for parameters analyzed in serum (s), that is, sodium (Na^+^), potassium (K^+^), creatinine and urea, was collected in plain tubes with a clotting activator and left to clot for a minimum of 30 min at room temperature. Then, samples were centrifuged at 1,469 *g* for 10 min at 4 °C. Plasma and serum were stored at −80 °C until analysis.

Basal blood samples for measurements of blood variables: CBC (complete blood count), Na^+^, K^+^, urea, glucose and insulin, were taken at rest in the fasting state between 7:30 and 8:00 a.m. The hematology (CBC) and biochemical (Na^+^, K^+^, urea, creatinine) tests were performed as described previously (Grandys et al., 2009). Plasma glucose was measured by enzymatic method (Vitros 950, Johnson and Johnson, USA) with the precision less than 1.5%. Insulin was measured by IRMA using the INS-IRMA kit (BioSource, Belgium). Analytical sensitivity for this measurement was 1 IU/ml and intra- and inter-assay CV were < 2.4% and 6.8%. For IRMA method the radioactivity of the samples were measured by using gamma scintillation counter (Wallac, Finland).

Blood samples (0.5 mL) for plasma lactate measurement [La^−^]_pl_ were taken before incremental exercise test (at rest, i.e.,  ∼2–3 h after light meal), at the end of each exercise intensity during incremental exercise just before the next increment of power output and at the end of the exercise protocol i.e., at exhaustion. [La^−^]_pl_ was analyzed by enzymatic method (Vitros 950, Johnson and Johnson, USA) as described previously (Majerczak et al., 2010) with precision less than 1.4%.

#### Plasma amino acids measurements

In the present study we aimed to determine the plasma amino acids concentrations (including BCAA) before exercise and during incremental exercise at strictly defined exercise intensities. The blood samples (1 mL) were taken at rest (pre-exercise) and at the power outputs corresponding to moderate exercise intensity (below LT), amounting in our study to 120 W (∼50% of VO_2max_), heavy exercise intensity (between LT and PO_max_), amounting in our study to 180 W (∼70% of VO_2max_), and at the maximal exercise intensity during the incremental exercise (at 100% of VO_2max_), amounting in our study to ∼264 vs 286 W, respectively before and after the training programme. In the light of the study by Felig & Wahren (1971), Bergström, Fürst & Hultman (1985) and Eriksson et al. (1985) a short-term exercise of low-to-moderate intensity should not lead to significant changes in BCAA concentration, but exercise of heavy intensity may affect their plasma concentrations. Total plasma α-amino acids concentration (including nonessential amino acids: alanine, arginine, asparagine, aspartate, glutamine, glutamate, glycine, proline, serine and tyrosine; essential amino acids: histidine, isoleucine, leucine, lysine, methionine, phenylalanine, threonine and valine) were determined using by Pico-Tag method. The Waters Chemistry Package for Free Amino Acid Analysis and the Ultrafiltration Sample Prep Kit (Waters, Milford, MA, USA) were used to perform the Pico-Tag method. 200 µL of plasma was mixed in a 1:1 ratio with the internal standard solution (methionine sulfone, 0.4mM in 0.1 M HCl) and filtrated using the Amicon MPS-1 ultrafiltration device. After filtration samples were reconstructed with drying solution consisting of a 2:2:1 mixture of methanol: 1 M sodium acetate: triethylamine (TEA), dried under vacuum and dissolved in 20 µL of derivatization reagent (7:1:1:1 solution of methanol: triethylamine (TEA): water: phenylisothiocyanate (PITC)). The derivatization process lasted for 20 min at room temperature and produced the corresponding phenylthiocarbonyl (PITC) derivatives. After derivatization samples were dried in vacuum and finally dissolved in 100 µL of Pico-Tag diluent (Waters, Milford, MA, USA) and injected. The column heater temperature was 46 °C, run time: 87 min, detector settings: 254 nm, 0.05 AUFS. The intra-assay coefficients of variation (CV) for leucine, isoleucine and valine was 0.42%, 0.79% and 1.19%, and the inter-assay CV was 2.04%, 3.53% and 2.27%, respectively. The method for plasma amino acids analysis performed in the present study was controlled by the European Research Network on Hereditary Metabolism (ERDNIM, MCA Lab, Netherlands).

### Data analysis

The plasma concentration of leucine ([Leu]_pl_), isoleucine ([Ile]_pl_), valine ([Val]_pl_) and the sum of those branched-chain amino acids ([∑BCAA]_pl_) during incremental exercise test were presented as absolute values measured at rest (pre-exercise), at 120 W (i.e., power output corresponding to ∼50% of VO_2max_), 180 W (i.e., power output corresponding to ∼70% of VO_2max_) and at PO_max_ (at 100% of VO_2max_) both before and after the training. Moreover, the changes in [Leu]_pl_, [Ile]_pl_, [Val]_pl_ and [∑BCAA]_pl_ during incremental exercise was presented in two exercise intensity domains: (a) when cycling in the moderate-intensity domain (below LT) and (b) when cycling in the heavy-intensity domain (above LT). The changes in BCAA concentration in the moderate-intensity domain was calculated as a difference (Δ_MI_) between concentration measured at 120 W (i.e. at power output corresponding to ∼50% of VO_2max_) and concentration measured at rest (pre-exercise). The change in BCAA concentration in the heavy-intensity domain (Δ_HI_) was calculated as a difference between concentration measured at PO_max_ (∼100% of VO _2max_) and value measured at 180 W (i.e., at power output corresponding to ∼70% VO_2max_). In addition exercise-induced changes in BCAA concentrations from rest to end of the incremental exercise was presented as a Δ_MAX_ i.e., the difference between the concentration measured at PO_max_ minus concentration at rest. The changes (Δ) in plasma BCAA concentration were presented both during incremental exercise performed before and after the training.

In the present study lactate threshold was defined as the highest power output (PO) above which [La^−^]_pl_ showed a sustained increase of lactate at least 0.5 mmol L^−1^in response to increasing PO by 30 W of every 3 min of incremental cycling (Zoladz, Rademaker & Sargeant, 1995; Zoladz et al., 2014).

Exercise-induced plasma volume changes were calculated using formula developed by *Van Beaumont (1972)*. Delta plasma volume (ΔPV) means difference between plasma volume before and after maximal incremental exercise. All values of plasma amino acids concentrations measured at intensity corresponding to PO_max_ were recalculated according to changes of plasma volume during incremental exercises using formula developed by *Berthoin et al. (2002)*.

The homeostatic model assessment of insulin resistance (HOMA-IR) was computed based on the fasting concentration of glucose and insulin using the following formula: HOMA-IR = [INS (μU mL^−1^) × GLU (mmol L^−1^)]/22.5, proposed by *Matthews et al. (1985)*.

### Statistical analysis

The results are presented as means and standard errors. The critical level of significance was set at *p* < 0.05. Statistical significance of differences for paired samples was tested using non-parametric Wilcoxon-signed-rank test and non-asymptotic, exact, two-sided *p*-values are presented.

In order to analyze the impact of exercise intensity (0, 120 W, 180 W, PO_max_) and training (before and after) on the [Leu]_pl_, [Ile]_pl_, [Val]_pl_, [∑BCAA]_pl_ and [La]_pl_ the data were analyzed using repeated measures 2-way ANOVA and planned comparisons were performed to identify significant differences between the groups. Moreover, repeated measures 2-way ANOVA was performed also to analyze the changes in [Leu]_pl_, [Ile]_pl_, [Val] _pl_, [∑BCAA]_pl_ and [La]_pl_ in the different intensity domains (Δ_MI_, Δ_HI_,  Δ_max_). The obtained *p-* values were corrected using the Holm-Bonferroni method. In order to quantify the dependence of ([Leu]_pl_, [Ile]_pl_, [Val]_pl_ and [∑BCAA]_pl_) on glucose homeostasis Spearman’s rank correlation coefficient was used. Statistical analysis was performed using statistical packet STATISTICA 10.0 (Stat Soft, Tulsa, OK., USA) and StatXact 9.0 (Cytel Software Corporation, Cambridge, MA, USA).

## Results

### Body composition and physical exercise capacity before and after the endurance training

A significant decrease in BM amounting to about 1.5%, was observed after endurance training (Table 1). No significant changes in absolute VO_2max_ was found (*p* = 0.19), however there was a tendency to higher relative VO_2max_ after the training program (45.6 ± 1.2 vs 47.3 ± 1.3 mL kg^−1^ min^−1^, respectively before and after training, ∼4% increase, *p* = 0.064).

**Table 1:** Body composition and exercise tolerance in the group of studied subjects before and after 5-weeks of the endurance training.

**Variables**	**Before training**	**After training**	***p*-value**
BM, kg	79.9 ± 2.7	78.7 ± 2.8	**0.046**
BF, %	15.7 ± 1.1	15.3 ± 0.9	0.19
VO_2max_, mL min^−1^	3627 ± 106	3701 ± 110	0.19
PO_max_, W	264 ± 8	286 ± 6	**0.004**
PO at LT, W	132 ± 8	129 ± 9	1.00
Time to exhaustion, min	26.4 ± 0.8	28.6 ± 0.6	**0.004**
HR_max_, bt min^−1^	190 ± 3	194 ± 2	**0.02**

**Notes.**

BM body mass BF% body fat percentage VO_2max_ maximal oxygen uptake PO_max_ power output reached at VO_2max_ PO at LT power output reached at lactate threshold HR_max_ maximum heart rate

Data are shown as mean ± S.E.M. Two-sided, exact, non-asymptotic *p*-values are presented (Wilcoxon signed-rank test).

The 5-week endurance training resulted in a significant increase (∼8%) in power output reached at VO_2max_, which was accompanied by the significant increase in time to exhaustion (Table 1). No significant changes in power output reached at lactate threshold (PO at LT) was observed (Table 1).

### Hematological and clinical chemistry parameters in the studied group of subjects before and after the endurance training

Basic hematological and clinical chemistry parameters measured at rest in a fasted state as well as plasma amino acids concentration i.e., total α-amino acids (including the sum of BCAA) and separately the sum of BCAA measured at rest in a fed state, before and after the training program are presented in Table 2. No significant difference (*p* > 0.05) in hematological parameters, electrolytes, glucose concentration and HOMA-IR were noticed after the training in the studied group, when compared to the before training values. Moreover, no significant difference in the total plasma amino acids concentrations as well as the sum of BCAA was observed after the training when compared to the values obtained before training.

**Table 2:** Hematological and clinical chemistry parameters in the studied group of subjects before and after 5-weeks of endurance training.

**Variable**	**Before training**	**After training**	***p*-value**
RBC, ×10^12^ L^−1^	5.07 ± 0.06	4.97 ± 0.08	0.29
WBC, ×10^9^ L^−1^	5.14 ± 0.33	5.76 ± 0.38	0.13
MCV, fL	87.5 ± 1.1	87.2 ± 1.2	0.26
Ht, %	43.9 ± 0.8	42.6 ± 0.9	0.14
Hb, g dL^−1^	15.4 ± 0.3	15.7 ± 0.6	0.94
[Na^+^]_*s*_, mmol L^−1^	141.2 ± 0.3	141.8 ± 0.5	0.23
[K^+^]_*s*_, mmol L^−1^	4.17 ± 0.09	4.17 ± 0.11	0.95
[Creatinine]_*s*_, mol L^−1^	92.8 ± 3.0	90.9 ± 2.2	0.56
[Urea]_*s*_, mmol L^−1^	5.27 ± 0.31	5.01 ± 0.35	0.51
[Glucose]_pl_, mmol L^−1^	5.28 ± 0.09	5.17 ± 0.07	0.26
[Insulin]_pl_, mIU L^−1^	10.9 ± 1.2	9.59 ± 1.24	0.70
HOMA-IR	1.43 ± 0.16	1.25 ± 0.16	0.70
[Σ*α* − *AA*]_pl_, µmol L^−1^	2973 ± 139	3,067 ± 150	0.77
[ΣBCAA]_pl_, µmol L^−1^	503 ± 24	561 ± 32	0.17

**Notes.**

RBC red blood cells WBC white blood cells MCV mean corpuscular volume Ht hematocrit Hb hemoglobin [Na^+^]_*s*_ serum sodium concentration [K^+^]_*s*_ serum potassium concentration [Creatinine]_*s*_ serum creatinine concentration [Urea]_*s*_ serum urea concentration [Glucose]_pl_ plasma glucose concentration [Insulin]_pl_ plasma insulin concentration HOMA-IR homeostatic model assessment-insulin resistance [Σ*α* − *AA*]_pl_ total plasma α-amino acids concentration [ΣBCAA]_pl_ total plasma branched-chain amino acids (sum of leucine, isoleucine and valine concentration)

Data are shown as mean ± S.E.M. Two-sided, exact, non- asymptotic *p*-values are presented (Wilcoxon signed-rank test).

### Plasma volume change during single bout of exercise before and after the endurance training

The average exercise-induced decrease in plasma volume (ΔPV) during the maximal incremental exercise test before training amounted to about 13% and did not differ significantly (*p* = 0.43) from its value during exercise performed after the training (ΔPV about 15%).

### The changes of plasma BCAA and lactate concentration during incremental cycling exercise

**Figure 1:**
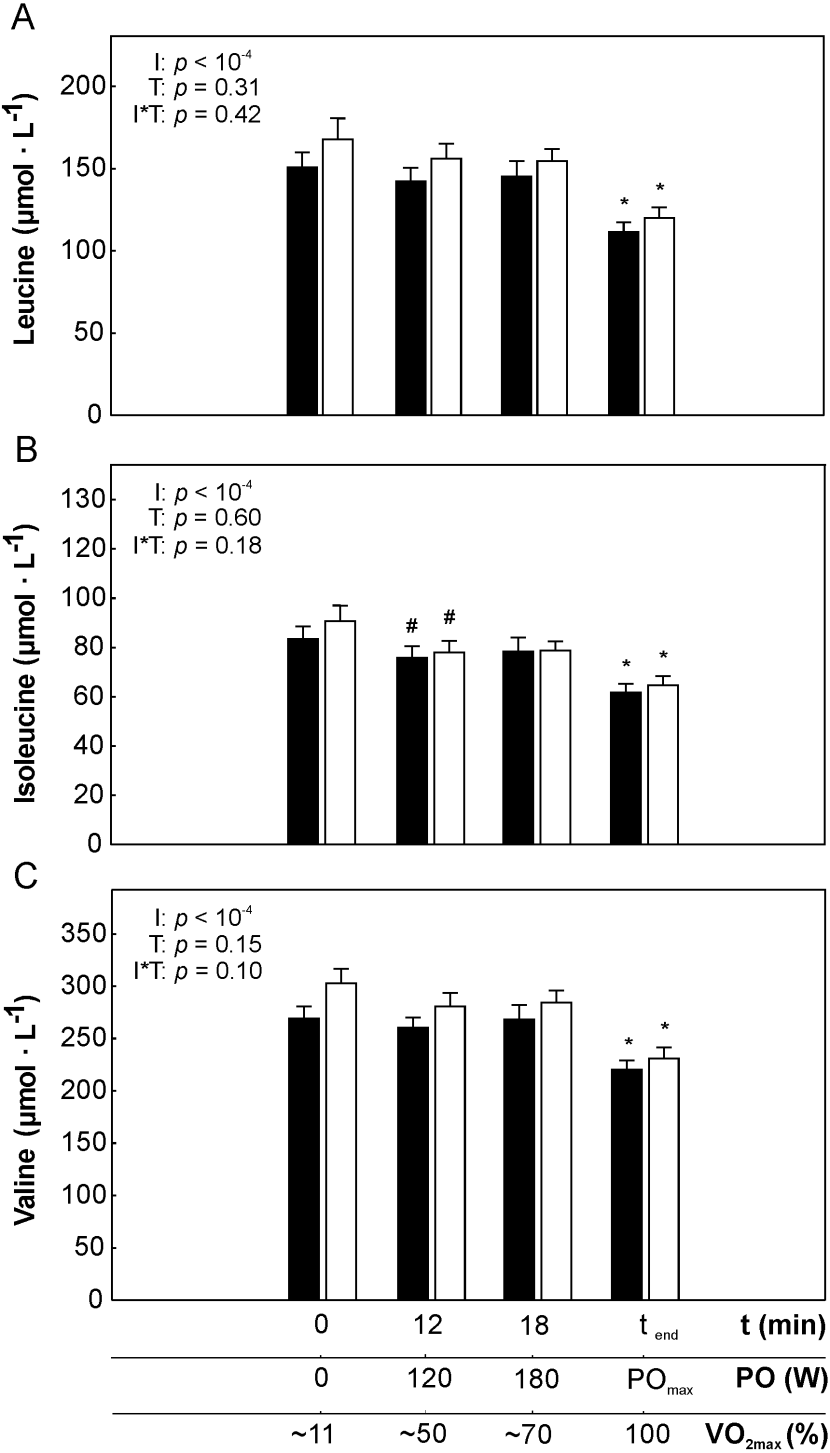
The changes in plasma branched-chain amino acids concentrations during incremental cycling exercise performed before and after the 5-weeks of the endurance training. The changes in plasma leucine (A), plasma isoleucine (B), plasma valine (C) during incremental exercise performed before (black bars) and after (white bars) the endurance training. The impact of factors: exercise intensity (I) and endurance training (T) on the analyzed variables is presented (repeated measures 2-way ANOVA, 2-sided *p*-value). Asterisk (*) represents significantly lower concentrations from 180 W, 120 W and from rest (*p* < 0.01) and hash (#) denotes significantly lower concentrations than at rest (*p* < 0.01). Data are shown as mean ±  S.E.M. Abbreviation: VO_2max_ – maximal oxygen uptake; PO_max_ – power output reached at 100% of maximal oxygen uptake. The percentage of VO_2max_ at the time zero represents the fraction of VO_2max_ measured in sitting position before exercise.

The changes in plasma leucine ([Leu]_pl_), isoleucine ([Ile]_pl_) and valine ([Val]_pl_) during incremental exercise performed before and after 5 weeks of the endurance training are presented in Figs. 1A–1C.

During incremental exercise performed before the training plasma valine concentration at 120 W (∼50% of VO_2max_) was not significantly different than at rest (*p* > 0.05, Fig. 1C). Plasma isoleucine concentration at 120 W was significantly lower than at rest (*p* < 0.01, Fig. 1B), whereas a tendency to lower [Leu]_pl_, was present at 120 W, when compared to rest value (*p* = 0.07, Fig. 1A). There was no significant difference (*p* > 0.05) in [Leu]_pl_, [Ile]_pl_ and[Val]_pl_ at intensity corresponding to 180 W (∼70% of VO_2max_) when compared to 120 W (∼50% of VO_2max_). However, at PO_max_(100% VO_2max_) plasma leucine, isoleucine and valine was significantly lower when compared to their values at 180 W (∼70% of VO_2max_). Moreover, plasma leucine, isoleucine and valine at PO_max_was significantly lower when compared with their value at rest (*p* < 0.01, Figs. 1A–1C).

**Figure 2:**
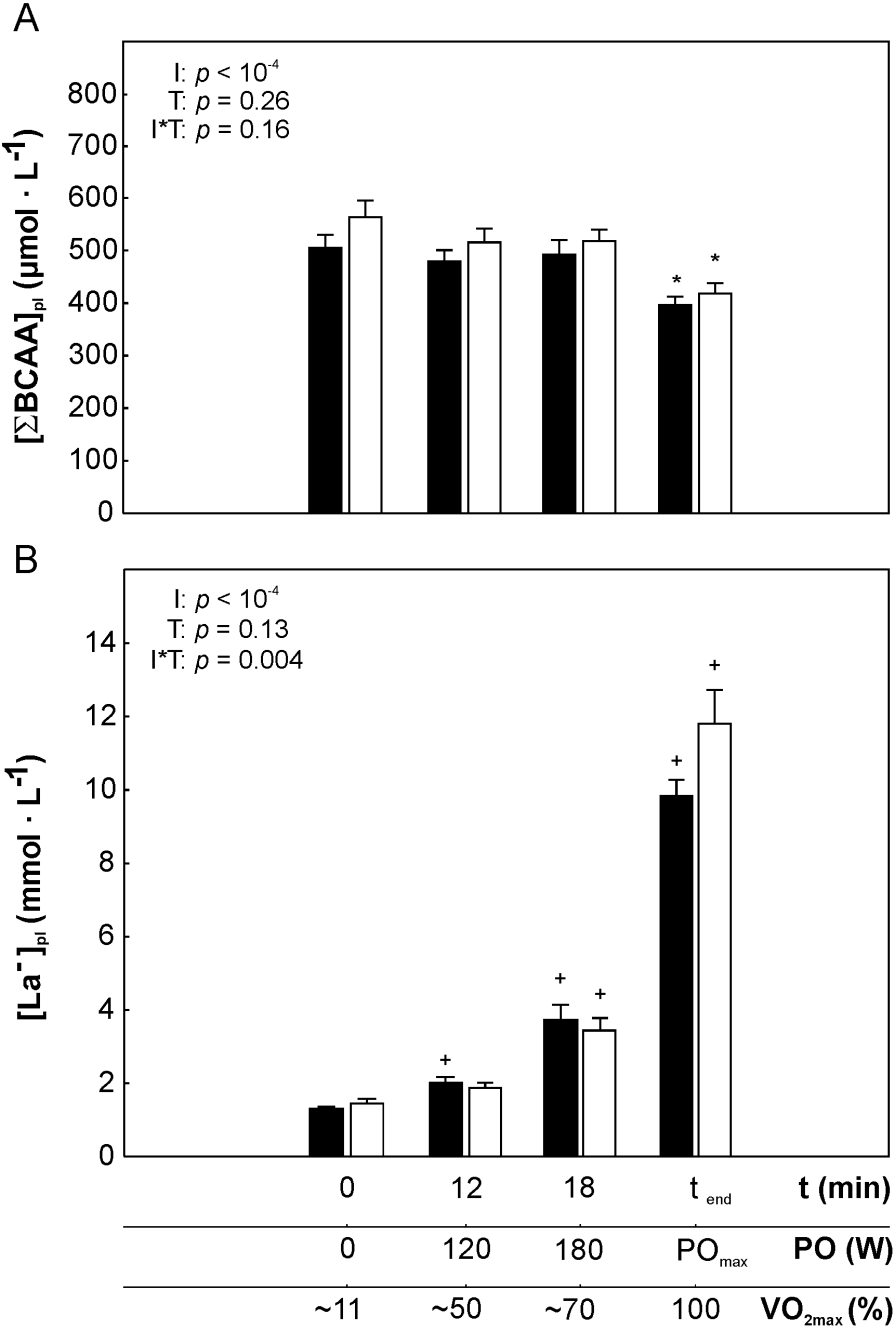
The changes in the sum of plasma branched-chain amino acids concentrations and plasma lactate concentration during incremental cycling exercise performed before and after the 5-weeks of the endurance training. The changes in the sum of plasma BCAA concentration ([∑ BCAA]_pl_, A) and plasma lactate concentration ([La^−^]_pl_), B) during incremental exercise performed before (black bars) and after (white bars) the endurance training. The impact of factors: exercise intensity (I) and endurance training (T) on the analyzed variables is presented (repeated measures 2-way ANOVA, 2-sided *p*-value). Asterisk (*) represents significantly lower concentrations from 180 W, 120 W and from rest (*p* < 0.01) and single cross (+) denotes significantly higher concentrations from the previous data point (*p* < 0.01). In the case of ([La^−^]_pl_ significant interaction between the two factors was observed (2-way ANOVA, I*T). Data are shown as mean ±  S.E.M. Abbreviation: VO_2max_ – maximal oxygen uptake; PO_max_ –  power output reached at 100% of maximal oxygen uptake. The percentage of VO_2max_ at the time zero represents the fraction of VO_2max_ measured in sitting position before exercise.

Since the changes of the [Leu]_pl_, [Ile]_pl_ and [Val]_pl_ during incremental exercise was similar for those three amino acids (Figs. 1A–1C), we have also analyzed the changes of the sum of BCAA ([∑BCAA]_pl_) during incremental exercise (Fig. 2A). We have found that [∑BCAA]_pl_ at 120 W was not significantly different than at rest, however a tendency to mild decrease when cycling in this intesity domain was observed (*p* = 0.08, Fig. 2A). No significant difference (*p* > 0.05) in [∑BCAA]_pl_ at intensity corresponding to 180 W (∼70% of VO_2max_) when compared to 120 W (∼50% of VO_2max_) was found, but at PO_max_ [∑BCAA]_pl_ was significantly lower than at 180 W ( ∼70% of VO_2max_) (*p* < 0.01, Fig. 2A).

The changes in plasma lactate concentration ([La^−^] _pl_) during incremental exercise is presented in Fig. 2B. An increase of power outputs from rest to 180 W was accompanied initially by a mild (up to 120 W) and further by a steeper (between 120 and 180 W) increase of plasma lactate concentration. However, exceeding 180 W (∼70% of VO_2max_) was accompanied by very steep increase of [La^−^]_pl_ which reached its maximum at PO_max_ (Fig. 2B).

The changes in [Leu]_pl,_ [Ile]_pl,_ [Val]_pl_ and [∑BCAA]_pl_ during incremental exercise performed after the endurance training was similar to that reported before the training i.e., the effect of exercise intesity was always statistically significant (Figs. 1A–1C). In case of lactate however, endurance training modified its concentration during exercise i.e., [La^−^]_pl_ after the traning was somewhat lower for intesities between 120 and 180 W, but visibly higher for PO_max_, when compared to pre-training levels (significant interaction I*T, *p* = 0.004, Fig. 2B).

**Figure 3:**
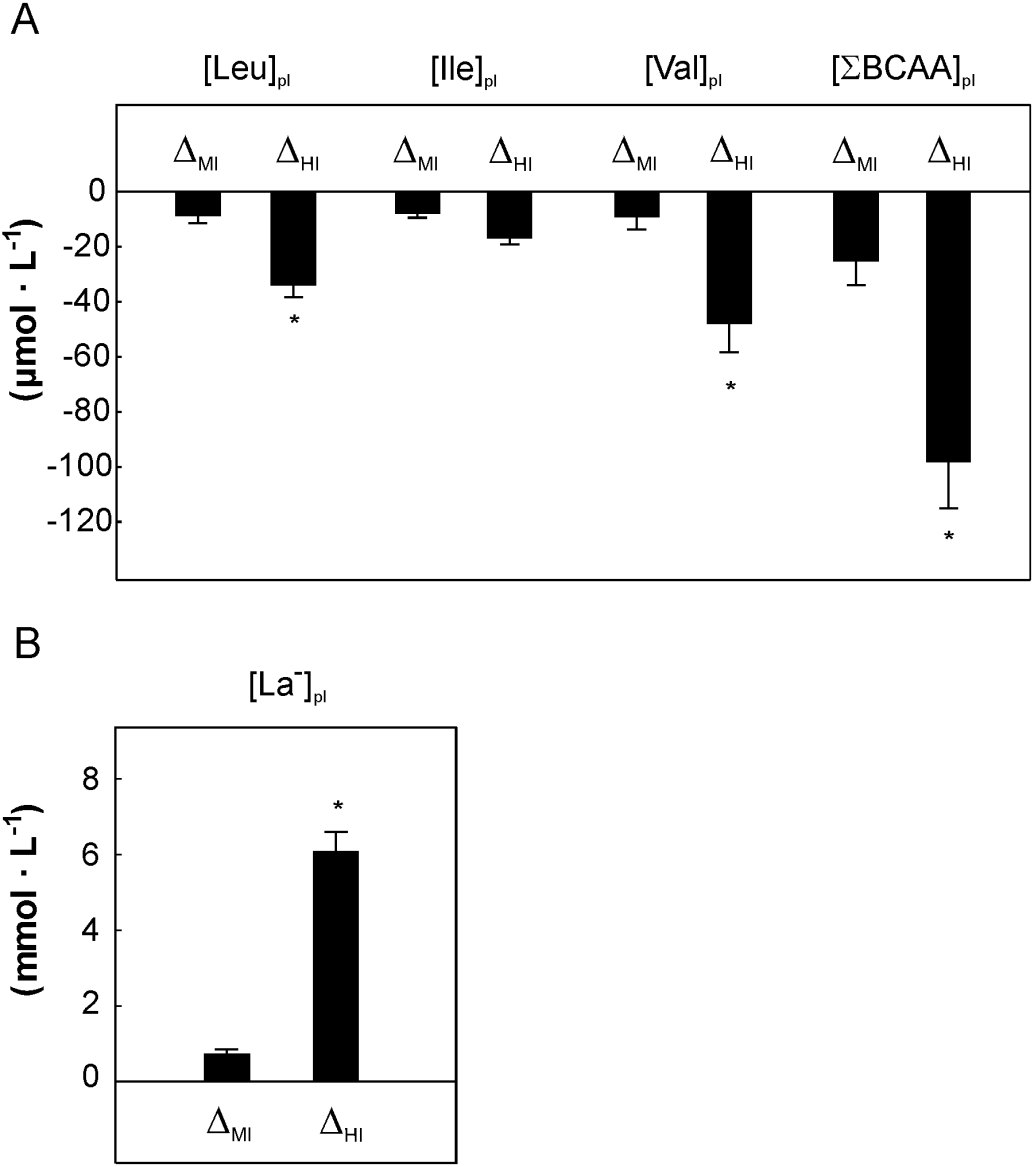
The impact of cycling performed before endurance training in the moderate (Δ_MI_) versus heavy (Δ_HI_) intensity domain on the plasma branched-chain amino acids (A) and plasma lactate concentration (B). Asterisk (*) represents significant difference from Δ_MI_ (*p* < 0.05, repeated measures 2-way ANOVA). Data are shown as mean ±  S.E.M. Abbreviations: [Leu]_pl_, plasma leucine concentration; [Ile]_pl_, plasma isoleucine concentration; [Val]_pl_, plasma valine concentration; [∑ BCAA]_pl_, the sum of plasma BCAA concentration, [La^−^]_pl_, plasma lactate concentration; Δ_MI_, the difference between value measured at ∼50% of VO_2max_ and value measured at rest; Δ_HI_, the difference between value measured at 100% of VO_2max_ (PO_max_) and value measured at ∼70% of VO_2max_.

**Figure 4:**
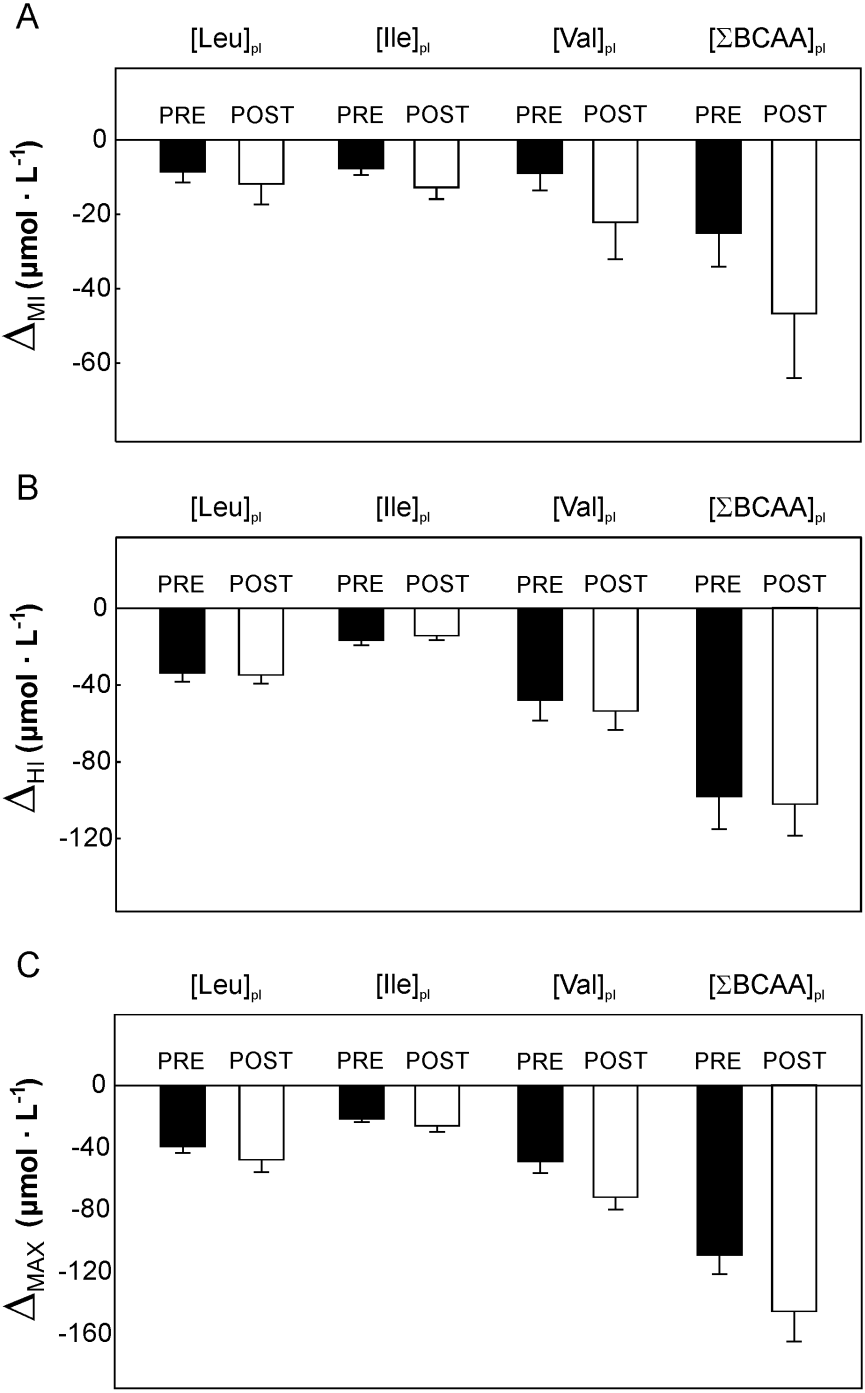
The impact of 5-weeks of the endurance training on the plasma branched-chain amino acids concentration during cycling in the varied intensity domains. The changes in plasma branched-chain amino acids concentration during cycling performed in the moderate-intensity domain (from rest up to 50% of VO_2max_ Δ_MI_, A), heavy- intensity domain (above ∼70% of VO_2max_, Δ_HI_, B) and during maximal incremental cycling (from rest to 100% of VO_2max_ - Δ_max_ C) before (Pre) and after (Post) the training. Data are shown as mean ±  S.E.M. Abbreviation: Abbreviations: [Leu]_pl_, plasma leucine concentration; [Ile]_pl_, plasma isoleucine concentration; [Val]_pl_, plasma valine concentration; [∑BCAA]_pl_, the sum of plasma BCAA concentration.

### The changes in plasma BCAA and lactate concentration during cycling performed in the moderate (Δ_MI_) versus heavy intensity (Δ_HI_) domain

When concerning the effect of exercise intensity on the BCAA changes during incremental exercise a greater decrease in [Leu]_pl_, [Val]_pl__,_[∑BCAA]_pl_ (*p* < 0.05) and tendency to greater decrease in [Ile]_pl_ (*p* = 0.08) have been observed during cycling in the heavy-intensity exercise domain i.e., above ∼70% of VO_2max_ up to 100% VO_2max_ (Δ_HI_) when compared to cycling in the moderate-intensity exercise domain i.e., below ∼50% of VO_2max_ (Δ_MI_) (Fig. 3A).

When considering the changes in plasma lactate concentration significantly greater increase in [La^−^]_pl_ was found during cycling in the heavy-intensity domain when compared to the moderate-intensity domain (*p* < 0.01 Fig. 3B).

### The impact of 5-weeks of the endurance training on the plasma BCAA concentration during incremental cycling exercise

No effect of 5-weeks of the endurance training (*p* > 0.05) on [Leu]_pl,_[Ile]_pl,_[Val]_pl_ and ∑[BCAA]_pl_ (Figs. 1A–1C and 2A) at rest and at appropriate exercise intensities (∼50% of VO_2max_, ∼70% of VO_2max_, and at intensity corresponding to 100% VO_2max_) have been observed. Moreover, no impact of endurance training on the changes (*p* > 0.05) in [Leu]_pl,_[Ile]_pl,_ [Val]_pl_ and ∑[BCAA]_pl_ during cycling performed in moderate (Fig. 4A) as well as in heavy (Fig. 4B) intensity domain were reported. Furthermore, no effect of 5 weeks of the endurance training (*p* > 0.05) on the maximal exercise-induced changes (Δ_max_) in [Leu]_pl_[Ile]_pl_ and ∑[BCAA]_pl_, was observed, except a clear tendency to a greater exercise-induced decrease in [Val]_pl_ during maximal incremental cycling performed after the training (*p* = 0.06, Fig. 4C).

### The impact of 5-weeks of the endurance training on the plasma lactate concentration during incremental cycling exercise

**Figure 5:**
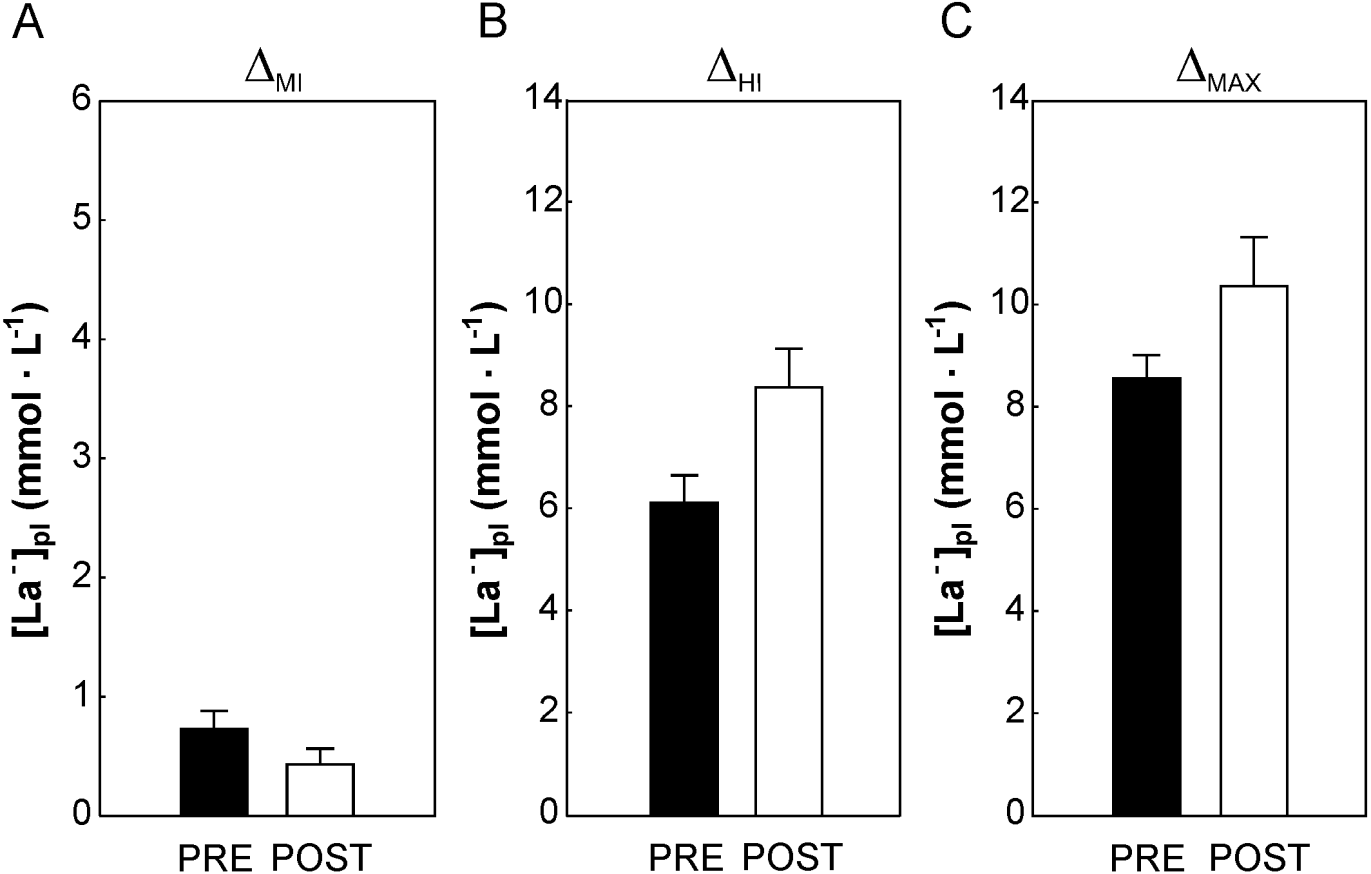
The impact of 5-weeks of the endurance training on the plasma lactate concentration during cycling in the varied intensity domains. The changes in plasma lactate concentration during cycling performed in the moderate-intensity domain (up to ∼50% of VO_2max_ − Δ_MI,_ A), heavy- intensity domain (above 70% of VO_2max_ - Δ_HI_, B) and during maximal incremental cycling (from rest to 100% of VO_2max_ - Δ_max,_ C) before (Pre) and after (Post) the training. Data are shown as mean ± S.E.M. Abbreviation: [La^−^]_pl_, plasma lactate concentration.

No effect of 5-weeks of endurance training (*p* > 0.05) on plasma lactate concentration at rest and during exercise (i.e., at ∼50% of VO_2max_, ∼70% of VO_2max_ and at 100% VO_2max_) have been observed (Fig. 2B). A tendency to lower increase in [La^−^]_pl,_ when cycling in the moderate-intesity domain was found (*p* = 0.07, Fig. 5A) after 5 weeks of the endurance training. On the other hand, a tendency to greater increase in [La^−^]_pl,_when cycling in the heavy-intesity domain (*p* = 0.08, Fig. 5B) as well as during maximal incremental cycling (*p* = 0.07, Fig. 5C) was reported after the training.

### Correlation analysis

No significant correlation (*p* > 0.05) between resting leucine, isoleucine, valine concentration and HOMA-IR both before and after the training was observed. A clear tendency to positive correlation between training-induced changes in the sum of BCAA [Σ BCAA]_pl_ at rest and training-induced change in basal glucose concentration was found (*r* = 0.61, *p* = 0.06).

## Discussion

The main finding of this study is that low-to-moderate intensity exercise (up to about 50% of VO_2max_) performed in young healthy men had essentially no significant impact on the plasma branched-chain amino acids concentration in relation to their levels at rest, however when cycling in heavy-intensity domain (i.e., above intensity corresponding to 70% of VO_2max_) a significant decrease in plasma BCAA concentration have been found. Interestingly, as presented in Fig. 2 the observed deeper decrease in plasma BCAA concentration (above ∼70% of VO_2max_) was accompanied by the onset of a faster increase in plasma lactate concentration. This suggests that the significant exercise-induced decrease in BCAA is linked to the acceleration of muscle glycogenolysis during exercise. We have also found that endurance training of moderate-intensity lasting 5 weeks resulting in significant enhancement of exercise tolerance had no significant impact on the exercise-induced changes in plasma BCAA concentrations.

### The changes in BCAA concentration during single bout of incremental exercise

In the present study we have analyzed the changes in plasma BCAA concentration during incremental exercise until exhaustion lasting about 30 min, which is a typical exercise test performed for the assessment of exercise tolerance *(Majerczak et al., 2014; Zoladz, Rademaker & Sargeant, 1995)*. We have found that a single bout of maximal incremental exercise performed in young, untrained, but physically active men was accompanied by a significant decrease (rest value versus intensity corresponding to VO_2max_) in leucine, isoleucine, valine (Figs. 1A–1C) and in the sum of BCAA (Fig. 2A). The results of the present study show that changes in BCAA concentration occur not only after prolonged, sustained exercise, as demonstrated earlier (Blomstrand, Celsing & Newsholme, 1988; Wagenmakers et al., 1989) but also are present after exercise of shorter duration (∼30 min). This might be a consequence of a significant increase in BCKDH activity in skeletal muscle during incremental exercise test as demonstrated by *Howarth et al. (2007)*.

Similar effect i.e., a significant decrease in BCAA concentration (rest versus intensity corresponding to VO_2max_) after single bout of whole body maximal graded exercise (an increase in power output 50 W every 3 min) in the group of young, healthy subjects has been observed in the study by Makhro et al. (2016). However, in the present study we have additionally analyzed the changes in BCAA concentration in varied exercise intensity domains during incremental exercise. We have found that short duration, low-to-moderate intensity exercise (i.e., up to ∼50% of VO_2max_) seems to have no significant impact on BCAA concentration (only a tendency to a mild decrease was observed below 50% of VO_2max_ and was followed by a plateau in BCAA in the range 50 and ∼70% of VO_2max_) whereas even short duration but heavy-intensity exercise (>70% of VO_2max_) decreases significantly circulating BCAA in young healthy men. Hence as consequence, we have observed significantly greater decrease in plasma BCAA during heavy-intensity cycling in comparison to cycling performed in the moderate-intensity domain (Fig. 3A). It should be mentioned that the changes in plasma leucine, isoleucine and valine concentration during single bout of incremental exercise found in the present study was similar for all three BCAA and also it was similar during exercise performed before and after the training (Figs.1A–1C).

The exercise-induced significant decrease in plasma BCAA concentration, when exceeding intensity corresponding to ∼70% of VO_2max_ might be a consequence of a greater rate of BCAA uptake by working skeletal muscles than splanchnic release when cycling in the heavy-intensity domain. Some authors postulated that higher exercise-induced amino acid delivery to muscles, including BCAA, results from blood flow redistribution during exercise with an involvement of epinephrine *(Zoladz et al., 2015; Zouhal et al., 2008)*. During heavy-intensity exercise skeletal muscles blood flow increases 100-fold when compared to resting conditions (Zoladz et al., 2015) and at the same time blood flow to liver is diminished by ∼50% when compared to its resting value (Bergeron et al., 2001). The blood flow redistribution might indeed explain an increase in BCAA uptake by skeletal muscles and a decrease in BCAA release from splanchnic bed (Brosnan, 2003; Zoladz et al., 2015) leading to a decrease of BCAA in the bloodstream.

Another factor, which might contribute to a significant decrease in BCAA during heavy-intensity exercise suggesting greater rate of their uptake by skeletal muscles could be related to the effect of hormones – insulin and epinephrine. BCAA appear to be the most sensitive amino acids to the insulin action, however based on the previous studies during incremental exercise of similar pattern no changes in insulin concentration or even an attenuation of plasma concentration of this hormone is observed *(Galbo, 1983; Zoladz et al., 2002)*. With regards to epinephrine, it has been demonstrated that epinephrine infusion exerts hypoaminoacidemic effect i.e., decreases all amino acids concentrations including BCAA and gluconeogenic amino acids in the bloodstream except alanine *(Del Prato et al., 1990)*. During incremental exercise plasma epinephrine concentration increases significantly when exceeding the intensity corresponding to about 50% of VO_2max_ (Galbo, 1983). Hence, an increase in epinephrine during graded exercise from intensity corresponding to 50% of VO_2max_ (Galbo, 1983) might explain the significant decrease in BCAA concentration observed in our study from intensity corresponding to ∼70% of VO _2max_ (Figs. 1A–1C and Fig. 2A). Moreover, it is well known that an increase in epinephrine concentration during incremental exercise (from ∼50% of VO_2max_) precedes the acceleration of glycogen utilization during exercise (Brooks & Mercier, 1994). It has been demonstrated that after crossing the so called ‘crossover point’ (carbohydrates/fatty acids utilization point), which in untrained subjects is localized at intensity corresponding to about ∼50% of VO_2max_, glycogen utilization increases disproportionally faster, leading to its depletion *(Brooks & Mercier, 1994)*. As a consequence of an intensification of glycogenolysis in skeletal muscles an increase in muscle and blood lactate accumulation occurs (Shulman, 2005). *Van Hall et al. (1996)* found that the rate of BCAA utilization (as reflected by an increase in percentage of active BCKDH complex) is higher (9-fold increase during exercise) in glycogen-depleted leg when compared to the leg with normal glycogen level (4-fold). This is consistent with the fact that BCAA can be used as an alternative source of energy for glycogen depleted muscles. In the present study, we observed the significant decrease in circulating BCAA concentration started at the exercise intensity corresponding to ∼70% of VO_2max_ (Fig. 2A), which was preceded by an intensification of plasma lactate accumulation (Fig. 2B). This coincidence clearly suggests that in untrained subjects an increased glycogen utilization in muscles during incremental exercise reflected by plasma lactate accumulation (Fig. 2B) precedes greater BCAA usage (Fig. 2A) as energy substrate during cycling in the heavy-intensity domain when compared to moderate exercise intensities.

Taking into account the role of systemic BCAA in the cardiometabolic risk, exercise at higher intensity (>70% of VO_2max_) in healthy, young individuals seems to be more effective in lowering of blood BCAA concentrations and in decreasing the cardiometabolic risk.

### The effect of 5-weeks of moderate-intensity endurance training on the physical capacity and plasma BCAA concentration

The endurance training applied in the present study was composed mainly of moderate-intensity exercise bouts (∼85% of training workloads was performed at intensity below lactate threshold). Such training intensities i.e., low to moderate are often prescribed as a part of therapy in cardiometabolic diseases which is a main cause of death in modern societies (Holloszy, 2005; Nystoriak & Bhatnagar, 2018). Training program performed by our subjects, amounting to a total of 20 exercise bouts, resulted in a significant increase in physical capacity as reflected by an increase in power output reached at VO_2max_ (by about 8%, *p* = 0.004) and by an increase in time to exhaustion (*p* = 0.004) after the training program (Table 1). An enhancement of physical capacity as judged by an increase in exercise duration and in enhancement of power generating capabilities after the applied training program (Table 1) was not accompanied by a significant changes in BCAA during exercise (Fig. 2A), except a clear tendency to greater decrease in plasma valine concentration during maximal incremental cycling performed after the training (*p* = 0.06, Fig. 4C). Moreover, we have observed no impact of endurance training on the circulating BCAA at rest and at appropriate power outputs (Figs. 1A–1C, 2A and 4A–4C).

The applied training program of moderate intensity lasting 5 weeks in the studied group of young healthy subjects did not change the insulin sensitivity as reflected by no changes in HOMA-IR (*p* > 0.05, Table 2). However, we have observed a clear tendency to positive correlation (*p* = 0.06) between training-induced change in the sum of BCAA at rest and training-induced changes in glucose concentration. This results support the suggested relation between higher plasma BCAA concentration with a decreased insulin sensitivity (Lotta et al., 2016; Wang et al., 2011). On the other hand, no significant correlation between training-induced changes in BCAA concentration at rest and changes in insulin resistance reflected by HOMA-IR has been found.

**Study limitations:** It should be underlined that the present study involved male subjects only in order to avoid a potential impact of sex differences. Therefore, the obtained results can be considered as representative for young healthy male only and the conclusion of the present study cannot be simply extrapolated to the females. Moreover, the number of subjects participating in this study was relatively small (10 individuals), nevertheless, this group was very homogeneous as described in the Method section. Furthermore, in the present study we have shown the changes of BCAA concentration at rest and at three exercise intensities—referring to moderate, heavy and severe intensity domain. It would be interesting to determine in the future the BCAA levels at all exercise stages during the maximal incremental exercise test. Another limitation of this study is lack of a control group. Taking into account the effect of circulating BCAA on insulin sensitivity and cardiometabolic health one should also consider to determine the impact of exercise in varied intensity domains on the BCAA concentrations in the group of participants at a more advanced age (e.g., over 50 years old) and patients including patients with cardiovascular disease and type 2 diabetes.

## Conclusions

We have concluded, that an incremental exercise performed in young healthy men has an impact on the plasma BCAA concentration in humans and in view of our results this effect depends on the exercise intensity domain. Namely, a short duration bouts of exercise (lasting about 12 min) of moderate intensity (up to about 50% of VO_2max_), as applied in e.g., cardiac rehabilitation or in recreational activities, has essentially no significant effect on the plasma BCAA concentrations in young healthy men. However, the exercise of high-intensity—above 70% of VO_2max_ (lasting only about 10 min) —leads to a pronounced decrease in plasma BCAA. This indicates that even short bouts of relatively high intensity exercise can decrease plasma BCAA in young healthy people, which implies that regular physical activity with implementation of heavy-intensity exercise bouts might be indeed more effective when consider an improvement of insulin sensitivity and prevention of type 2 diabetes. Concomitantly, endurance training of moderate-intensity lasting 5 weeks, resulting in significant increase in exercise tolerance had no effect on the exercise-induced changes in plasma BCAA concentrations. Taking into account the role of the BCAA in cardiometabolic diseases the effect of physical activities on the plasma BCAA requires further studies, especially in the group of patients with cardiometabolic risk.

## Additional Information and Declarations

### Data Availability

The following information was supplied regarding data availability: Data are available at Jagiellonian University Repository: Gawędzka, A., Grandys, M., Duda, K., Zapart-Bukowska, J., Zoladz, J. A., & Majerczak, J. (2020). Data to figures and tables. Repozytorium Uniwersytetu Jagiellońskiego. https://ruj.uj.edu.pl/xmlui/handle/item/244739.
